# The coronary artery calcium score is linked to plasma cholesterol synthesis and absorption markers: Brazilian Longitudinal Study of Adult Health

**DOI:** 10.1042/BSR20201094

**Published:** 2020-07-02

**Authors:** Valéria Sutti Nunes, Isabela M. Bensenor, Paulo A. Lotufo, Marisa Passarelli, Edna Regina Nakandakare, Eder Carlos Rocha Quintão

**Affiliations:** 1 Laboratorio de Lipides, LIM-10, Hospital das Clinicas HCFMUSP, Faculdade de Medicina, Universidade de Sao Paulo, Sao Paulo, SP, Brazil; 2 Center for Clinical and Epidemiologic Research, University of São Paulo, São Paulo, Brazil

**Keywords:** campesterol, carotid artery intima-media thickness, cholesterol, coronary artery calcium, desmosterol

## Abstract

It is controversial whether atherosclerosis is linked to increased intestinal cholesterol absorption or synthesis in humans. The aim of the present study was to relate atherosclerosis to the measurements of plasma markers of cholesterol synthesis (desmosterol, lathosterol) and absorption (campesterol, sitosterol). In healthy male (*n*=344), non-obese, non-diabetics, belonging to the city of São Paulo branch of the Brazilian Longitudinal Study of Adult Health (ELSA-Brasil), we measured in plasma these non-cholesterol sterol markers, together with their anthropometric, dietary parameters, traditional atherosclerotic risk factors, and blood chemistry, coronary arterial calcium score (CAC), and ultrasonographically measured common carotid artery intima-media thickness (CCA-IMT). Cases with CAC>zero had the following parameters higher than cases with CAC = zero: age, waist circumference (WC), plasma total cholesterol (TC), low-density lipoprotein-cholesterol (LDL-C), and non-high density lipoprotein-cholesterol (non HDL-C). Plasma desmosterol and campesterol, duly corrected for TC, age, body mass index (BMI), waist circumference (WC), hypertension, smoking, and the homeostasis model assessment-insulin resistance (HOMA-IR) correlated with CAC, but not with CCA-IMT**.** The latter related to increased age, BMI, waist circumference (WC), and systolic blood pressure (SBP). Plasma HDL-C concentrations did not define CAC or CCA-IMT degrees, although in relation to the lower tertile of HDL-C in plasma the higher tertile of HDL-C had lower HOMA-IR and concentration of a cholesterol synthesis marker (desmosterol). Present work indicated that increased cholesterol synthesis and absorption represent primary causes of CAD, but not of the common carotid artery atherosclerosis.

## Introduction

Plasma non-cholesterol synthesis and absorption sterols have been utilized as markers of cardiovascular disease (CVD) [1–6]. Accordingly, compared with controls, established cardiovascular disease cases, not on lipid lowering medication, presented plasma sterols significantly higher as cholesterol absorption markers and lower as cholesterol synthesis markers [3,4,6–9]. A review on the subject [10] suggested that in most of the studies increased level of the markers of cholesterol absorption is a risk factor for coronary artery disease (CAD). Nonetheless, according to a review of population studies the inverse relationship between cholesterol absorption and synthesis does not appear to be a frequent finding in all publications [11]. However, in another study, common carotid artery intima-media thickness (CCA-IMT) correlated with serum cholesterol and sterol markers of cholesterol synthesis, but was weakly negatively correlated with the cholesterol absorption markers [12]. Furthermore, patients with cardiovascular disease had elevated markers of synthesis and decreased markers of intestinal cholesterol absorption in relation to controls, but also were older and had greater body mass index (BMI) [8]. Consequently, the controversy over the role in CVD of the synthesis and absorption markers of cholesterol remains. This controversy is even more provocative due to the well-established relationship of diabetes mellitus (DM) with atherosclerotic CVD [13,14]. In this regard, various publications show controversial results on plasma non-cholesterol sterols as markers of cholesterol metabolism in DM. For instance, no difference in cholesterol absorption was reported in Type1 DM, with and without CAD [15], although low synthesis and high absorption of cholesterol characterized Type 1 DM [16]. On the other hand, insulin resistance is associated with increased cholesterol synthesis, and decreased cholesterol absorption in normal blood glucose men [17], and sterol markers of cholesterol synthesis in plasma cluster with clinical and laboratory markers of obesity and insulin resistance [18]. On the contrary, significant relationship between insulin sensitivity and indices of increased cholesterol synthesis and of decreased cholesterol absorption has been reported [19].

In order to decide on the relevance of the plasma non-cholesterol sterols as markers of synthesis and intestinal absorption of cholesterol in atherosclerosis, we measured cross-sectionally these sterols in 344 participants belonging to the city of São Paulo branch of the Brazilian Longitudinal Study of Adult Health (ELSA-Brasil), a multi-cohort study in Brazil. We also measured their anthropometric, dietary parameters, including traditional atherosclerotic risk factors, such as smoking, blood pressure (BP), alcohol ingestion, BMI, plasma lipids, glucose, insulin and high-sensitivity C reactive protein (hsCRP), together with the CCA-IMT and coronary artery calcium score (CAC).

## Materials and methods

ELSA-Brasil is an ongoing prospective cohort of 15,105 civil servants aged 35–74 years in six state capital cities in Brazil. Baseline assessments occurred between 2008 and 2010. Further details of this cohort were described elsewhere [20–22]. In brief, all active or retired civil servants of six academic institutions were eligible for the study. Participants selected according to their occupations were classified as unskilled, technical/clerical and faculty and professional staff, permitting a gradient of socioeconomic position across the sample. The ELSA‐Brasil protocol was approved by the National Committee for Ethics in Research (CONEP Brazil: 976/2006) and by Committee for Ethics in Research of Hospital Universitário da Universidade de São Paulo (CEP-HU/USP: 659F/06). All participants signed a written informed consent form. Exclusion criteria were current or recent pregnancy, intention to quit working at the institution, severe communication impairment, and residence outside of a study center’s metropolitan area. Anthropometric measurements were obtained using standard techniques. BP was measured using a validated oscillometric device (Omron HEM 705CPINT). Three measurements were taken at 1-min intervals, and the mean of the two last BP measurements was considered as the BP value. Hypertension was defined as the use of medications to treat hypertension, a systolic blood pressure (SBP) ≥140 mmHg or a diastolic blood pressure (DBP) ≥90 mmHg. All participants from the city of São Paulo underwent a CAC examination performed with a 64-detector computed tomographic scanner (Brilliance 64; Philips Healthcare, Best, The Netherlands). After the scout images, each patient also underwent an electrocardiogram-gated prospective calcium score examination with a tube potential of 120 kV and a tube current adjusted to body habitus. Images were reconstructed in 2.5-mm slice thickness using standard filtered back projection. CAC was expressed as Agatston units, and the percentile was evaluated in a blinded fashion by an experienced cardiologist using semiautomatic software (Calcium Scoring, Philips Workstation). Coronary artery calcium severity was further categorized as zero or > zero [23]. CCA-IMT was assessed using ultrasound Toshiba scanner (Aplio XG™) and measured in the far wall of a predefined carotid segment 1cm in length below the carotid bifurcation. Mean maximal CCA-IMT from both common carotid arteries was used [24]. Exclusion criteria were female gender, diabetes, treatment with lipid-lowering drugs or using drugs for chronic diseases (except for 33% of hypertensive cases), self-report of previous CVD, and BMI ≤ 18.5 and ≥ 35 kg/m^2^, totaling 445 participants from the city of São Paulo chosen for this report. Considering that morbidly obese individuals present a high cardiovascular risk, they were excluded from our investigation.

A food frequency questionnaire was utilized to identify the alimentary pattern including the 61 most commonly consumed foods representing 86% of total energy intake plus other foods that, based on expert opinion, were commonly consumed by today’s urban population. This questionnaire provided the daily intake of total fat and fat types (saturated, monounsaturated and polyunsaturated) and cholesterol [25].

### Determination of sterols in plasma by GCMS

The plasma samples selected from the Bio-bank of the Central Laboratory of the ELSA-Brasil project at the Hospital of the University of São Paulo were sent to the Lipids Lab (LIM10), Hospital das Clinicas, HC-FMUSP, University of São Paulo, for analyses of non-cholesterol sterol markers of intestinal cholesterol absorption (campesterol and sitosterol), and of cholesterol synthesis (desmosterol and lathosterol), by gas chromatography (GC), coupled to the mass spectrophotometer (MS) (Shimadzu GCMS-QP2010 plus -Kyoto, Japan), using version 2.5 of the GCMS solution software.

Plasma samples (200 μl) were mixed with the internal standard (5α-cholestane:1 μg) and hydrolyzed with 1 ml KOH diluted in ethanol (1 mol/l) at 60°C for 1 h. Water (1 ml) was added and extraction done with hexane twice (2 ml). The hexane was evaporated and the sterols were derivatized with silanizing solution, consisting of pyridine (100 μl), BSTFA (N,O-bis(trimethylsilyl) trifluoroacetamide: 100 μl), 1% TMCS (trimethylchlorosilane) (1:1, v/v) (Supelco 33155-U) and incubated for 1 h at 60°C. The derivatized sample (1 μl) was injected into the chromatograph by the automatic injector in split injection mode with split ratio 1:3 and the injector temperature maintained at 280°C. Separation was performed on the Restek capillary column (100% dimethyl polysiloxane-Rxi13323), 30 m long, internal diameter 0.25 mm, for 30 min. Helium was the mobile phase with constant linear velocity (45.8 cm/s), and the oven temperature maintained at 280°C. The mass spectrometer operates in impact electron mode at an ionization voltage of 70 eV with the temperature of the ion source and the interface at 300°C. Sterol ions were identified on the mass spectrometer utilizing the Single Ion Monitoring (SIM) method (Table 1). GC-MS/SIM sterol was quantified by comparison with the peak areas, and the mass spectra of the standard ion curve corrected by the internal standard [26].

**Table 1:** Sterol ions monitored in the mass spectrometer by the SIM method

	Ion target	Ion 1	Ion 2
5α-cholestane	217	109	149
Desmosterol	253	351	143
Lathosterol	255	213	458
Campesterol	129	343	382
Sitosterol	129	357	396

### Statistical analysis

Statistical analyses were performed by GraphPad Prism version 4.00, with significance level *P*<0.05. Data as mean ± standard deviation (S.D.) and as median and range are presented according to their parametric or non-parametric distributions, respectively. Comparisons between groups utilized an unpaired Student’s *t* test and the Mann–Whitney test for CAC.

Logistic regression models (95% confidence intervals) was used to study the association of tertiles of plasma campesterol, sitosterol, desmosterol and lathosterol with CAC = zero vs. CAC > zero after multivariate adjustment for continuous variables such as age, BMI, WC, plasma TC and HOMA-IR and categorical variables such as hypertension (yes or no), and smoking (never, past or current). We used generalized linear models to study the association of the same markers of absorption and synthesis with CCA-IMT after the same multivariate adjustment.

## Results

Table 2 shows baseline clinical characteristics of the participants, including CAC and CCA-IMT values, blood chemistry, plasma lipids, non-cholesterol sterol markers of cholesterol synthesis and absorption, and food lipid pattern. Percent frequencies were for smoking (20%), alcohol use ≥ 210g/week (40%), and presence of hypertension (33%). The diet composition did not differ between cases and controls.

**Table 2:** Baseline demographic, clinical characteristics, blood chemistry, plasma non-cholesterol sterols, and food lipid patterns of the study sample

	*n*	Mean ± S.D.
Age (years)	344	56 ±4
BMI (kg/m²)	344	25 ± 3
WC (cm)	344	90 ± 8
SBP (mmHg)	344	123 ± 15
DBP (mmHg)	344	77 ± 10
CAC (Agatston)	344	78 ± 2180 (0–2737)
CCA-IMT (mm)	336	0.640 ± 0.124
**Blood chemistry**		
Fasting glucose (mg/dl)	344	106 ± 8
Glucose - 2 h (mg/dl)	341	125 ± 30
HbA1C (%)	344	5.21 ± 0.54
Insulin (µUI/ml)	344	6.65 ± 5.88
Insulin - 2 h (µUI/ml)	341	48 ± 40
HOMA-IR	344	1.74 ± 1.54
Creatinine (mg/dl)	344	1.01 ± 0.18
Sodium (mEq/l)	344	144 ± 3
Potassium (mEq/l)	344	4.6 ± 0.50
AST (U/l)	344	24.6 ± 8.9
ALT (U/l)	344	27.2 ± 10.2
Gamma GT (U/l)	344	32.9 ± 15.5
Uric acid (mg/dl)	344	6.3 ± 1.3
TSH (µUI/ml)	344	1.92 ± 1.16
Hs CRP (mg/l)	344	2.16 ± 5
**Plasma lipid profile (mg/dl)**		
TC	344	217 ± 36
LDL-C	344	138 ± 30
Non-HDL-C	344	164 ± 34
TG	344	134 ± 74
HDL-C	344	52 ± 14
TG/HDL-C ratio	344	2.84 ± 1.94
**Plasma non-cholesterol sterols (µg/ml)**		
Desmosterol	299	0.512 ± 0.284
Lathosterol	337	1.123 ± 0.589
Campesterol	337	1.368 ± 0.960
Sitosterol	337	2.249 ± 1.381
**Plasma non-cholesterol sterols ratio (µg/mg cholesterol)**		
Desmosterol	299	0.240 ± 0.139
Lathosterol	337	0.527 ± 0.278
Campesterol	337	0.643 ± 0.460
Sitosterol	337	1.054 ± 0.652
**Cholesterol synthesis-absorption ratio**		
Lathosterol/campesterol	337	1.217 ± 1.025
Lathosterol/sitosterol	337	0.675 ± 0.537
**Food lipid pattern (total amount /day)**		
Lipids (g)	344	94 ± 40
SAT(g)	344	31 ± 14
MUFA (g)	344	30 ± 13
PUFA (g)	344	24 ± 10
Cholesterol (mg)	344	335 ± 179

Abbreviations: ALT, alanine aminotransferase; AST, aspartate aminotransferase; BMI, body mass index; CAC, coronary arterial calcium; CCA-IMT, carotid artery intima-media thickness; DBP, dastolic blood pressure; HbA1c (%), hemoglobin A1c; HOMA-IR, homeostasis model assessment-insulin resistance; HDL-C, high-density lipoprotein cholesterol; hsCRP, high-sensitivity C-reactive protein; LDL-C, low-density lipoprotein cholesterol; MUFA, monounsaturated fatty acids; PUFA, polyunsaturated fatty acids; SAT, saturated fatty acids; SBP, systolic blood pressure; TC, total cholesterol; TG, triglycerides; TSH, thyroid-stimulating hormone; WC, waist circumference.

It was also observed that compared with the cases with CAC = zero, those with CAC > zero had age, WC modestly higher, although not differing as to BMI, BP, glucose, hemoglobin A1c (HbA1c), insulin, and homeostasis model assessment-insulin resistance (HOMA-IR) (Table 3). Higher values for TC, LDL-C, non-HDL-C and CCA-IMT occurred in CAC > zero. As for the plasma non-cholesterol sterols markers of cholesterol synthesis and absorption, only desmosterol, a synthesis marker expressed per plasma volume or adjusted for the plasma TC concentration (plasma non-cholesterol sterols ratio) was markedly greater in cases of CAC >zero than in cases of CAC = zero.

**Table 3:** CAC categories (CAC = zero vs. CAC > zero, median 61 (1-2737 Agatston units), baseline clinical characteristics, plasma lipid profiles, non-cholesterol sterols, glucose, insulin, and plasma non-cholesterol sterols

	CAC = zero		CAC > zero	
	*n*	Mean ± S.D.	*n*	Mean ± S.D.
Age (years)	178	55 ± 3	166	57 ± 4^b^
BMI (kg/m²)	178	25 ± 3	166	25 ± 3
WC (cm)	178	89 ± 8	166	91 ± 8^a^
SBP (mmHg)	178	123 ± 15	166	123 ± 16
DBP (mmHg)	178	78 ± 10	166	77 ± 10
CCA-IMT (mm)	173	0.621 ± 0.114	163	0.661 ± 0.130^a^
Fasting glucose (mg/dl)	178	106 ± 8	166	107 ± 8.3
Glucose - 2 h (mg/dl)	178	124 ± 29	166	127 ± 30
HbA1C (%)	178	5.20 ± 0.54	166	5.20 ± 0.48
Insulin (µUI/ml)	178	6.54 ± 6.27	166	6.70 ± 5.39
Insulin - 2 h (µUI/ml)	178	46 ± 36	166	51 ± 43
HOMA IR	178	1.71 ± 1.65	166	1.77 ± 1.4
TC (mg/dl)	178	211 ± 36	166	223 ± 34^b^
LDL-C (mg/dl)	178	134 ± 32	166	142 ± 28^a^
Non-HDL-C (mg/dl)	178	159 ± 36	166	170 ± 32^a^
Triglycerides (mg/dl)	178	127 ± 66	166	143 ± 80
HDL-C (mg/dl)	178	52 ± 13	166	53 ± 14
TG/HDL-C	178	2.72 ±1.78	166	2.97 ± 2.10
**Plasma non-cholesterol sterols (µg/ml)**				
Desmosterol	151	0.457 ± 0.227	148	0.568 ± 0.324^b^
Lathosterol	172	1.084 ± 0.589	165	1.163 ± 0.588
Campesterol	172	1.305 ± 0.882	165	1.433 ± 1.033
Sitosterol	172	2.211 ± 1.362	165	2.289 ± 1.404
**Plasma non-cholesterol sterols ratio (µg/mg cholesterol)**				
Desmosterol	151	0.220 ± 0.109	148	0.260 ± 0.162^a^
Lathosterol	172	0.519 ± 0.258	165	0.535 ± 0.298
Campesterol	172	0.632 ± 0.432	165	0.655 ± 0.488
Sitosterol	172	1.066 ± 0.662	165	1.042 ± 0.643

Abbreviations: BMI, body mass index; CAC, coronary arterial calcium; CCA-IMT, carotid artery intima-media thickness; DBP, diastolic blood pressure; HbA1c (%), hemoglobin A1c; HDL-C, high-density lipoprotein cholesterol; HOMA-IR, homeostasis model assessment-insulin resistance; LDL-C, low-density lipoprotein cholesterol; SBP, systolic blood pressure; TC, total cholesterol; TG, triglycerides; WC, waist circumference. Unpaired *t* test, CAC = zero vs. CAC > zero, 61 (median = 1–2737), a: *P*<0.05; b: *P*<0.001.

To evaluate the parameters that influence the atherosclerotic lesion in the carotid arteries, we compared the degrees of CCA-IMT below and above the 75th percentile. The degree of CAC determined the intensity of the coronary lesion, and CCA-IMT did have sufficient sensitivity to disclose the intensity of the atherosclerotic process as shown by the elevated CAC (Table 4). The CCA-IMT score varied according to age, WC, BMI, SBC, as well as to total cholesterol, LDL-C, and non-HDL-C concentrations in plasma.

**Table 4:** CCA-IMT categories IMT < 75th percentile (0.705, mean 0.583 ± 0.073 mm) vs. IMT ≥75th percentile (0.710, mean 0.803 ± 0.085 mm), baseline clinical characteristics, plasma lipid profiles, non-cholesterol sterols, glucose, insulin, and plasma non-cholesterol sterols

	CCA-IMT <75th percentile		CCA-IMT ≥75th percentile	
	*n*	Mean ± S.D.	*n*	Mean ± S.D.
Age (years)	248	56 ± 3	88	57 ± 4^a^
BMI (kg/m²)	248	25 ± 3	88	26 ± 2^a^
WC (cm)	248	89 ± 8	88	91 ± 7^a^
SBP (mmHg)	248	122 ± 15	88	126 ± 18^a^
DBP (mmHg)	248	76 ± 10	88	78 ± 10
CAC (Agatston)^c^	248	0 (0–2737)	88	19 (0–1461)^b^
Fasting glucose (mg/dl)	248	106 ± 9	89	107 ± 7
Glucose - 2 h (mg/dl)	247	125 ± 29	89	124 ± 30
HbA1C (%)	247	5.2 ± 0.5	89	5.2 ± 0.5
Insulin (µUI/ml)	247	6.3 ± 5.1	89	7.3 ± 7.6
Insulin - 2 h (µUI/ml)	244	48 ± 38	89	50 ± 45
HOMA IR	247	1.71 ± 1.65	89	1.77 ± 1.4
TC (mg/dl)	248	215 ± 36	88	224 ± 33^a^
LDL-C (mg/dl)	248	135 ± 31	88	145 ± 29^a^
Non-HDL-C (mg/dl)	248	161 ± 35	88	172 ± 32^a^
TG (mg/dl)	248	131 ± 72	88	146 ± 79
HDL-C (mg/dl)	248	53 ± 14	88	50 ± 11
TG/HDL-C	248	2.74 ± 1.88	88	3.11 ± 2.07
**Plasma non-cholesterol sterols (µg/ml)**				
Desmosterol	213	0.506 ± 0.283	78	0.533 ± 0.287
Lathosterol	241	1.112 ± 0.628	88	1.169 ± 0.617
Campesterol	241	1.351 ± 0.906	88	1.453 ± 1.113
Sitosterol	241	2.246 ± 1.389	88	2.292 ± 1.383
**Plasma non-cholesterol sterols ratio (µg/mg cholesterol)**				
Desmosterol	213	0.239 ± 0.140	78	0.243 ± 0.142
Lathosterol	241	0.525 ± 0.295	88	0.525 ± 0.235
Campesterol	241	0.645 ± 0.444	88	0.657 ± 0.513
Sitosterol	241	1.069 ± 0.668	88	1.029 ± 0.619

Abbreviations: BMI, body mass index; CAC, coronary arterial calcium; CCA-IMT, carotid artery intima-media thickness; DBP, diastolic blood pressure; HbA1c (%), hemoglobin A1c; HDL-C, high-density lipoprotein cholesterol; HOMA-IR, homeostasis model assessment-insulin resistance; LDL-C, low-density lipoprotein cholesterol; SBP, systolic blood pressure; TC, total cholesterol; TG, triglycerides; WC, waist circumference. Unpaired *t* test and the Mann–Whitney test for CAC, IMT < 0.705, vs. IMT ≥ 0.710: a, *P*<0.05; b, *P*<0.001; c = median (range).

Clinical and blood chemical parameters relating coronary arterial CAC and CCA-IMT with the lower as compared with the higher tertile of plasma cholesterol concentrations are shown in Table 5. It shows that clinical and blood chemical parameters relate to CAC but not to CCA-IMT in the lower as compared with the higher tertile of plasma cholesterol concentrations. CAC score is more dependent on the TC concentration than CCA-IMT.

**Table 5:** Clinical and blood chemical parameters relating CAC and CCA-IMT with the lower as compared to the higher tertile of plasma cholesterol concentrations

	Cholesterol <200 mg/dl		Cholesterol >231 mg/dl	
	*n*	Mean ± S.D.	*n*	Mean ± S.D.
Age (years)	117	55 ± 3	118	56 ± 4^a^
BMI	117	25 ± 3	118	25 ± 2
WC (cm)	117	89 ± 8	116	90 ± 8
SBP (mmHg)	117	124 ± 15	116	124 ± 18
DBP (mmHg)	117	78 ± 10	116	78 ± 12
CAC (Agatston)^c^	117	0(0–1148)	118	14 (0–2737)^a^
CCA-IMT	112	0.624 ± 0.114	116	0.657 ± 0.130
TC (mg/dl)	117	180 ± 17	118	255 ± 24^b^
TG (mg/dl)	117	113 ± 59	118	153 ± 82^b^
HDL-C	117	48 ± 13	118	57 ± 14^b^
LDL-C (mg/dl)	117	110 ± 19	118	168 ± 23^b^
Desmosterol (µg/mg)	102	0.195 ± 0.103	98	0.277 ± 0.168^b^
Lathosterol (µg/mg)	116	0.448 ± 0.219	115	0.592 ± 0.312^b^
Campesterol (µg/mg)	116	0.515 ± 0.344	115	0.719 ± 0.525^b^
Sitosterol (µg/mg)	116	0.899 ± 0.520	115	1.145 ± 0.734^b^

Abbreviations: BMI, body mass index; CAC, coronary arterial calcium; CCA-IMT, carotid artery intima-media thickness; DBP, diastolic blood pressure; HbA1c (%), hemoglobin A1c; HDL-C, high-density lipoprotein cholesterol; HOMA-IR, homeostasis model assessment-insulin resistance; LDL-C, low-density lipoprotein cholesterol; SBP, systolic blood pressure; TC, total cholesterol; TG, triglycerides; WC, waist circumference. Unpaired *t* test and the Mann–Whitney test for CAC; cholesterol <200 mg/dl vs. cholesterol >231 mg/dl: a, *P*<0.05; b, *P*<0.001; c = median (range).

After multivariate adjustments for age, BMI, WC, BP, TC concentration, smoking and HOMA-IR index, desmosterol (Table 6) and campesterol (Table 7) were associated with CAC, although not with CCA-IMT.

**Table 6:** Odds ratio (OR) and 95% confidence intervals (95% CI) for the association of tertiles of desmosterol (cholesterol synthesis marker) with CAC

CAC (Agatston)		
Desmosterol (µg/ml)	OR (95% CI)	*P* value
1st tertile	1.0 (reference)	
2nd tertile	1.739 (0.937–3.228)	0.080
3rd tertile	3.241 (1.700–6.179)	<0.001

Abbreviation: CAC, coronary arterial calcium. Multivariate adjustment for age, BMI, WC, hypertension, TC, smoking and HOMA-IR.

**Table 7:** Odds ratio (OR) and 95% confidence intervals (95% CI) for the association of tertiles of campesterol (cholesterol absorption marker) with CAC

CAC (Agatston)		
Campesterol (µg/mL)	OR (95% CI)	*P* value
1st tertile	1.0 (reference)	
2nd tertile	1.553 (0.859–2.805)	0.145
3rd tertile	1.858 (1.020–3.387)	0.043

Abbreviations: CAC, coronary arterial calcium. Multivariate adjustment for age, BMI, WC hypertension, TC, smoking and HOMA-IR.

## Discussion

The present study aimed at elucidating the role of plasma non-cholesterol sterol absorption and synthesis markers of cholesterol as parameters of CV risk of atherosclerotic disease in the prospective ELSA-Brasil study. The investigation was limited to the male gender in view atherosclerotic cardiovascular disease is more precocious than in the female gender.

Due to the homogeneous food habit of the population investigated, we assumed that the intake of phytosterols did not vary significantly among the participants; this must be taken into account because diminishing the absorption of the dietary cholesterol, moderate doses of phytosterols in the usual diet could have influenced the profile of lipids in the plasma [27–29].

Differences shown in plasma sterols as related to CAC and CCA-IMT degrees indicate that the simultaneous presence of coronary and carotid artery lesions may not follow identical chronology [24,30–32]. In this regard, there are risk factors capable of having variable impact on carotid intima-media thickness and on coronary arteries [32,33]. In support of this interpretation, in the elderly CCA-IMT relates more strongly to stroke than CAC, although CAC is also an important predictor of stroke [34]. Furthermore, a high CCA-IMT could reflect not only a pathological intima thickening as the first step of the atherosclerosis process, but also the hypertrophy of the media layer [34].

Plasma desmosterol as marker of cholesterol synthesis varied with CAC but not with CCA-IMT grade. This may have occurred because the degree of atherosclerosis in the carotid and in the coronary depends on specific risk factors. For example, carotid artery lesion may depend on the arterial hypertension mainly, while the coronary artery disease may depend more on elevated LDL-C, which in turn may relate to increased cholesterol synthesis and intestinal absorption.

Concerning the plasma sterol markers, we also found discrepancies of our results compared with the literature. An investigation had shown inverse correlation between CCA-IMT and cholesterol synthesis as measured by endogenous sterol fecal excretion, and direct correlation with the intestinal absorption of cholesterol [7]. However, we noticed their participants were overweight (mean BMI = 28.1), and 13% were on statins. Other population investigations correlating CAD with sterol markers of cholesterol absorption directly, and with sterol markers of cholesterol synthesis inversely, might be biased for including diabetic and obese participants [5,35]. Our work associating CAC with increased cholesterol synthesis, indicated by the high plasma desmosterol concentration, agrees in part with another study in which CCA-IMT related to the sterol synthesis marker, and not to the sterol absorption marker [12]. However, in another study on the same German population, these authors also reported a relationship with CVD that is direct with plasma markers of intestinal absorption, and inverse with plasma markers of cholesterol synthesis [4]. The reason for their discrepant results is not known, but we suggest here that it may be due to their studies not having been prospective, or else, that the first study was on the measurement of CCA-IMT, while the second one referred to CVD cases that were identified by coronary angiography, peripheral vascular disease and cerebrovascular disease, the latter reported by clinical history.

The prospective Framingham study results obtained for the plasma markers of synthesis and absorption also differed from our investigation: patients of both genders with CVD and/or carotid stenosis above 50%, but not taking lipid-lowering drugs, showed a significant increase in cholesterol absorption markers, with decrease in cholesterol synthesis markers [2]. Nonetheless, the latter study differed from ours in some important aspects. In Framingham, participants were correctly matched for age, BMI, plasma lipoprotein profile and smoking frequency, but the study was contaminated by confounders such as the presence of diabetes, glucose intolerance, higher frequency of hypertension treated cases and mean BMI (28.7 kg/m^2^) in the CVD groups than in controls. Therefore, it included overweight and metabolic syndrome cases while in our population the average BMI was 25 kg/m^2^, and excluded diabetes and morbid obese cases. This is an important point because it has been reported significantly increased cholesterol synthesis or decreased cholesterol absorption in metabolic syndrome and diabetes [3] related to insulin resistance [19]. Furthermore, it is important to take into account the known relationship of insulin resistance with the CCA-IMT that occurs even in non-diabetic population [36], and the relation of metabolic syndrome parameters with elevation of sterol synthesis markers [1]. Insulin resistance had not been reported in the Framingham investigation although it is known that markers of cholesterol synthesis cluster with clinical and laboratory markers of obesity and insulin resistance [18,37], and the latter is associated with increased cholesterol synthesis and decreased cholesterol absorption even in normoglycemic men [17]. In a Framingham investigation, CAD was associated with the cholesterol synthesis marker (squalene) concentration that was low in women and high in men. In the latter the markers of absorption had no predictive value for coronary disease, but nonetheless also included obese and metabolic syndrome cases [3]. On the other hand, in a meta-analysis, plasma concentrations of plant sterols, known as markers of intestinal cholesterol absorption, failed to relate with CVD [38].

Our results indicate that the process of atherosclerosis is diffuse because CAC and CCA-IMT are simultaneous as previously reported by others [39]. In other words, atherosclerosis results from diverse metabolic defects whose simultaneity would contribute to its premature development. Cases with CAC > zero and with CCA-IMT ≥ 75th percentile present relationships in common to various risk factors for atherosclerotic cardiovascular disease (Tables 3 and 4), such as age, BMI, WC, TC, LDL-C, and non-HDL-C. However, some parameters are more specific for the atherosclerosis risk in a certain vascular territory: serum desmosterol for CAC and SBP for CCA-IMT. It is notable that the direct cause of atherosclerotic disease is the elevation of the TC concentration. As a function of this fact, we examined the behavior of the plasma synthesis and absorption markers according to tertiles of plasma TC concentration (Table 5). It is quite evident that cholesterol concentration related to the age of the participants, to both markers of cholesterol synthesis and absorption, and to CAC, but not to CCA-IMT.

However, both desmosterol and campesterol related to CAC when properly corrected for several parameters, including age [40] (Tables 6 and 7). This result seems somehow surprising because synthesis and absorption of cholesterol are processes that often oppose one another. However, as shown here they may simultaneously be present in the population contributing to the elevation of blood cholesterol and, consequently, act together to increase the risk for atherosclerotic cardiovascular disease.

It is important to compare our research with the European LURIC and YFS cohort studies [6]. That publication attributed on the ABCG8 and ABO alleles did not correlate risk of cardiovascular disease with cholesterol synthesis, measured by the lathosterol/cholesterol ratio, but only with the elevation of intestinal cholesterol absorption, measured by the cholestanol/cholesterol ratio. Nevertheless, the authors did not publish the other important marker of synthesis (desmosterol/cholesterol ratio), which contrarily to lathosterol, in our work related with CAC.

Plasma HDL-C concentration was stratified by tertiles below 45 mg/dl and above 55 mg/dl (Table 8). In the higher HDL-C tertile, we found modestly higher values as age, TC, and LDL-C and lower BMI, WC, TG, TG/HDL-C, insulin, 2 h insulin and glucose, and HOMA-IR, compatible with diminished insulin resistance. In this group, it was observed diminished plasma desmosterol concentration and desmosterol/cholesterol ratio, indicating reduced cholesterol synthesis rate, as previously published [41], but that did not define the degree of CAC or CCA-IMT. This finding represents much less relevant role of HDL-C compared with LDL-C as a risk factor for atherosclerotic disease.

**Table 8:** Comparison of the HDL-C concentration mean tertiles (HDL-C> 55 mg/dl vs HDL-C <45 mg/dl) according to age, BMI, WC, BP, TC, LDL-C, TG, glucose, insulin, HOMA-IR, CAC, CCA-IMT, and plasma markers of cholesterol synthesis and absorption

	HDL-C< 45 (mg/dl)		HDL-C > 55 (mg/dl)	
	*n*	mean ± S.D.	*n*	mean ± S.D.
Age (years)	112	55 ± 3	114	56 ± 4^a^
BMI (kg/m²)	112	26 ± 2	114	24 ± 3^b^
WC (cm)	112	92 ± 7	114	86 ± 8^b^
SBP (mmHg)	112	124 ± 14	114	122 ± 16
DBP (mmHg)	112	78 ± 10	114	76 ± 11
CAC (Agatston)^c^	112	0–961	114	0–2737
CCA-IMT (mm)	112	0.645 ± 110	114	0.620 ± 0.113
TC (mg/dl)	112	201 ± 29	114	226 ± 38^b^
LDL-C (mg/dl)	112	129 ± 27	114	139 ± 32^a^
Non-HDL-C (mg/dl)	112	161 ± 28	114	159 ± 38
TG (mg/dl)	112	165 ± 84	114	101 ± 52^b^
TG/HDL-C	112	4.21 ± 2.27	114	1.56 ± 0.90^b^
Fasting glucose (mg/dl)	112	108 ± 8	114	106 ± 9
Glucose - 2 h (mg/dl)	111	133 ± 30	112	121 ± 30^b^
HbA1C (%)	112	5.3 ± 0.6	114	5.2 ± 0.5
Insulin (µUI/ml)	112	8.0 ± 5.8	114	4.8 ± 3.9^b^
Insulin - 2 h (µUI/ml)	111	58.2 ± 47.2	112	37.1 ± 28.8^b^
HOMA IR	112	2.12 ± 1.55	114	1.26 ± 1.04^b^
**Plasma non-cholesterol sterols (µg/mL plasma cholesterol)**				
Desmosterol	95	0.536 ± 0.306	95	0.505 ± 0.283
Lathosterol	111	1.132 ± 0.606	110	1.124 ± 0.606
Campesterol	111	1.371 ± 0.991	110	1.348 ± 0.804
Sitosterol	111	2.145 ± 1.308	110	2.359 ± 1.408
**Plasma non-cholesterol sterols (µg/mg plasma cholesterol)**				
Desmosterol	95	0.271 ± 0.160	95	0.224 ± 0.128^a^
Lathosterol	111	0.571 ± 0.321	111	0.500 ± 0.238
Campesterol	111	0.685 ± 0.488	111	0.616 ± 0.381
Sitosterol	111	1.071 ± 0.643	111	1.063 ± 0.615

Abbreviations: BMI, body mass index; CAC, coronary arterial calcium; CCA-IMT, carotid artery intima-media thickness; DBP, diastolic blood pressure; HbA1c (%), hemoglobin A1c; HDL-C, high-density lipoprotein cholesterol; HOMA-IR, homeostasis model assessment-insulin resistance; LDL-C, low-density lipoprotein cholesterol; SBP, systolic blood pressure; TC, total cholesterol; TG, triglycerides; WC, waist circumference. Unpaired *t* test and the Mann-Whitney test for CAC, IMT <0.705, vs. IMT ≥0.710: a, *P*<0.05; b, *P*<0.001. c = median (range).

Present work indicates that increased cholesterol synthesis and absorption characterized, respectively, as high concentration of desmosterol and campesterol when properly corrected for age, BMI, WC, hypertension, TC, smoking and HOMA-IR represent primary causes of CAD, but not of the common carotid artery atherosclerosis. The latter preferably related to the increase in systolic blood pressure. The present study is limited to measuring degrees of atherosclerosis found in two procedures such as CCA and CCA-IMT, but does not measure effective end points indicative of the disease such as mortality from acute myocardial infarction and stroke.

## Perspectives

- The present study was carried out with the purpose of evaluating the importance of measuring sterols in plasma markers of synthesis and intestinal cholesterol absorption in identifying the incidence and importance of atherosclerotic lesions measured in the common carotid as compared with the coronary arteries in non-diabetic non-obese adult male subjects.
- The coronary artery calcium score (CAC), not the common carotid-intima thickness (CCA-IMT), is secondary to simultaneously increased cholesterol synthesis and intestinal cholesterol absorption.
- Consequently, the development of the coronary artery calcium score likely preferably is preventable by drugs that simultaneously block the increased cholesterol synthesis and intestinal absorption rates.

## Abbreviations

BMI: body mass index
CAC: coronary arterial calcium
CCA-IMT: carotid artery intima-media thickness
DBP: diastolic blood pressure
HbA1c: hemoglobin A1c
HDL-C: high-density lipoprotein cholesterol
HOMA-IR: homeostasis model assessment-insulin resistance
LDL-C: low-density lipoprotein cholesterol
SBP: systolic blood pressure
TC: total cholesterol
TG: triglycerides
WC: waist circumference
